# Impact of tobacco cessation education on behaviors of nursing undergraduates in helping smokers to quit smoking

**DOI:** 10.18332/tid/139024

**Published:** 2021-07-14

**Authors:** Li Zhang, Xian Long Huang, Tao Ye Luo, Li Jiang, Mei Xue Jiang, Han Yan Chen

**Affiliations:** 1 College of Nursing, Chongqing Medical University, Chongqing, China; 2 Respiratory Medicine Department, The First Branch of the First Affiliated Hospital of Chongqing Medical University, Chongqing, China; 3 Clinical Epidemiology and Biostatistics Department, Children's Hospital of Chongqing Medical University, Chongqing, China; 4 Respiratory Medicine Department, The First Affiliated Hospital of Chongqing Medical University, Chongqing, China; 5 Disease Prevention and Healthcare Department of Healthcare Center of Xiejiawan Subdistrict of Jiulongpo District, Chongqing, China

**Keywords:** 5As, evidence-based practice, tobacco dependence treatment, nursing students, smoking cessation

## Abstract

**INTRODUCTION:**

Smoking continues to be a significant public health issue, but nursing students do not receive sufficient training on tobacco cessation education. Integrating the 5As behaviors for tobacco cessation into a compulsory course could improve nursing students’ skills and increase their clinical behaviors for assisting patients in quitting smoking. The aim of this study was to evaluate the impact of evidence-based tobacco cessation education on the perceptions and behaviors of nursing students who are assisting patients to quit smoking.

**METHODS:**

A prospective single-group design was used to evaluate the perceptions and behaviors of 626 senior nursing students enrolled in an education program, at three time points: baseline, 3 months post education, and 6 months post education. Data were collected, before and after the tobacco cessation education, using assessment tools for knowledge, attitudes, and the 5As behaviors for assisting patients to quit smoking.

**RESULTS:**

A total of 572 senior students completed the baseline survey, 289 students completed the survey 3 months post education, 348 students completed the survey 6 months post education, and 285 students completed all three surveys. Knowledge and self-efficacy of tobacco cessation were improved dramatically (p<0.05) after the education program, compared with the baseline survey. At 6 months post education, compared with at 3 months post education, nursing students reported more interventions of asking, advising, assessing, assisting, and arranging smokers to quit smoking (p<0.05).

**CONCLUSIONS:**

The integration of tobacco cessation education into compulsory courses could improve clinical skills and enhance the behaviors of nursing students for assisting patients to quit smoking.

## INTRODUCTION

The evidence suggests that global tobacco users do not obtain the help they need to quit smoking^1^. The Framework Convention on Tobacco Control (FCTC) developed by the World Health Organization (WHO) and Article 14 of its guidelines stresses the need to improve global medical professionals’ abilities to deliver tobacco dependence treatment through various educational strategies, providing many approaches to tobacco treatment to members, including China, therefore strengthening their ability to assist patients in quitting smoking. The International Council of Nurses and the International Society of Nurses in Cancer Care both support the WHO FCTC on tobacco cessation and have asked nurses to implement brief smoking cessation interventions for inpatients^2^. Nursing students are future nurses, and education about tobacco cessation could promote their responsibility for smoking cessation and improve their knowledge and skills for assisting patients in quitting smoking. However, the Global Health Professions Student Survey (GHPSS) found that no more than 40% of nursing students have received formal tobacco cessation education^3^. China, the largest producer and consumer of cigarettes in the world, currently has 316 million smokers, and approximately 1 million smokers die of tobacco-related diseases each year^4^. If China does not adopt quick and effective tobacco cessation interventions to control tobacco prevalence, China could experience a dismally large number of deaths caused by tobacco.

To prevent the adverse effects of tobacco on people, the Chinese government has taken active measures to control smoking by adopting the WHO FCTC and trying to fulfil the obligations to ban smoking in public, establish smoke-free schools and hospitals, publicize the harm of tobacco through media, and implement a Healthy China Action plan requiring medical professionals to be non-smoker models and actively assist patients in quitting smoking.

The China Clinical Smoking Cessation Guidelines (Guidelines)^5^ clearly require that all medical professionals should practice the following 5A behaviors for helping patients to quit smoking: 1) Ask patients about their smoking history, 2) Advise patients who smoke to quit, 3) Assess patients’ readiness to quit, 4) Assist patients who want to quit to stop smoking, 5) Arrange follow-up for smokers who take action to quit smoking and assist with the challenges of smoking cessation.

However, medical professionals in China do not universally demonstrate the 5As behaviors for assisting patients in quitting smoking. The first step to smoking cessation is to ask patients about their smoking history. At the same time, only 63.1% of professionals reported a high frequency of asking about smoking status at a tertiary A hospital in Beijing, which was one of the first smoke-free hospitals in China^6^. A survey in Beijing and Hefei found that only 63.9% of nurses frequently asked patients about their smoking history^7^. The lack of tobacco cessation behaviors can be attributed to a lack of knowledge and skills due to the lack of corresponding education in the undergraduate nursing curriculum^8,9^.

Tobacco cessation education is an effective approach for improving the knowledge and skills of medical professionals^10^. Tobacco cessation education for nurses has been shown to increase the 5As behaviors for assisting smokers in quitting smoking^11–14^. Tobacco cessation training as entry-level education for medical students has been shown to increase the frequency of behaviors for assisting clients to quit smoking^15^. Studies have shown that evidence-based tobacco cessation education dramatically improved nursing students' intention to participate in smoking cessation, improved their skills, and enhanced their confidence^16–19^. Sohn^16^ implemented tobacco cessation education in an elective nursing course at a South Korean university, including a 2-hour lecture on cessation strategies and a 3-hour simulation, and found a significant improvement in the self-efficacy of participants for assisting patients to quit smoking. Shishani^17^ adopted the content of a tobacco cessation program into compulsory courses and improved the clinical skills of nursing students for assisting patients to quit smoking through online education and simulation practice in various settings. Schwindt^18^ thought it was important to increase confidence and motivation in order to assist patients to quit smoking and developed a tobacco cessation program which was designed based on the self-determination theory (SDT) to integrate tobacco cessation into a course on Mental Health Nursing/Mental Health Care. This program reported significant improvement in motivation for assisting patients with mental health problems to quit smoking. Along with the development of information, the blended learning of traditional face-to-face interaction and e-learning have demonstrated flexibility in practical teaching. Choi^19^ administered blended teaching of tobacco cessation education for nursing students and enhanced their self-motivation and self-efficacy for assisting patients to quit smoking.

The above-mentioned studies have shown that different formats of tobacco cessation education for nursing students can enhance knowledge, attitudes, self-efficacy, and skills for smoking cessation. However, few studies have focused on assessing the 5As behaviors of students for assisting patients in quitting smoking post education. According to the theory of knowledge, attitude, and practice, those skills are the basis but not the necessary and sufficient conditions of behaviors. The impact of tobacco cessation education on the 5As behaviors of nursing students for assisting patients to quit smoking is not completely known. As the largest producer and consumer of cigarettes, China has reported limited tobacco cessation education for nursing students^9,20^, possibly because the disease-centered curriculum model has ignored the instruction of this important public health skill^21,22^. To investigate the impact of tobacco cessation education on the behaviors of nursing students, as well as to improve students’ smoking cessation skills, and to develop more medical professionals for assisting patients in quitting smoking in a clinic setting, in this study, we integrated tobacco cessation education into a compulsory course. We assessed the impact on the knowledge, attitudes, and self-efficacy, as well as the behaviors of nursing students for assisting patients in quitting smoking.

## METHODS

### Study design

This study followed a one-group study design and used an objective sampling method to provide tobacco cessation education for 626 senior nursing students. We assessed changes in knowledge, attitudes, selfefficacy, and the 5As behaviors for assisting patients in quitting smoking, at 3 and 6 months post-education. This study was approved by the Ethics Committee of the First Affiliated Hospital of Chongqing Medical University (No. 2019-157), and students were informed that their participation was voluntary. A total of 572 senior nursing students participated in the baseline survey, and 285 students completed all three surveys, i.e. baseline, 3 months post-education, and 6 months post education.

### Course procedure and content

Students received 6 hours of instruction on tobacco cessation, including a 2-hour didactic session, a 2-hour online self-learning session, and a 2-hour scenario simulation session. The didactic lecture introduced tobacco harm and the importance of tobacco cessation, regulations and measures for tobacco cessation, health-related theories such as the transtheoretical model of health behavior change, methods and skills of health education, and the 5As behaviors and 5Rs for assisting patients to quit smoking.

Students were required to study the online resources within one month after the didactic lecture, including the epidemiology of tobacco use, nicotine pharmacology and addiction principles, smoking cessation drugs, brief theories of smoking cessation intervention, interventions at different stages of smoking cessation, 5As behaviors for assisting patients to quit smoking, and tools for aiding smoking cessation. The online learning contents were mainly from the Guidelines^5^ and a short intervention manual for smoking cessation (Manual)^23^ which was compiled by the Tobacco Control Office of CDC of China with the help of the WHO, which were usually employed for training and self-study of medical professionals. After 3 years of implementation, the Manual^23^ was revised and improved, and published. It was used to popularize brief tobacco cessation interventions and to meet the needs of medical professionals and smokers about tobacco cessation. The Manual^23^ involved a PowerPoint for training, demonstration videos, teaching cases, test questions, simple tools for tobacco cessation intervention, and questionnaires for teaching assessment, all free and accessible on the website of the Tobacco Control Office of CDC (http://www.notc.org.cn/). The online learning session was processed on the e-learning website of the Chongqing Medical University (http://e-learning.cqmu.edu.cn/meol/index.do) with the functions of learning resource uploading, learning monitoring, questioning and answering (Q and A), online quiz, and learning statistics. Lecturers supervised the learning process and provided timely help when problems presented.

The students were required to complete an online test after the learning process. When they passed the test (grade ≥60), they attended the simulation session. Students were provided with 9 cases for role playing, which included possible cases in clinical settings at different stages of readiness to quit, i.e. ‘unwilling to quit’, ‘willing but not ready to quit’, ‘ready to quit’, and ‘quitter’. Those cases involved patients of different ages, with various diseases, as well as different cultures. Each class was divided into 4 groups with 4–5 students, i.e. one patient, one to two family members, one nurse, and one observer. When the simulation finished, the students summarized their positive and negative learning experiences, by completing a brief report focusing on reflective learning.

### Faculty

Four teachers participated in the study, one senior teacher from the community health service center who was engaged in a community smoking control service, two primary teachers of smoking cessation in clinical nursing work, and one senior teacher who administered community nursing education and conducted tobacco control research on nursing students. Before the learning process, the teachers discussed the form and content of teaching, referring to the Guidelines^5^ and the Manual^23^, to ensure that the students received homogeneous smoking cessation education.

### Assessment tools

The knowledge and attitudes of tobacco cessation, as well as self-efficacy of assisting patients to quit smoking were assessed by surveys completed prior to education, 3 months post education, and 6 months post education, as well as the post-education 5As behaviors. The survey was developed with reference to the Manual^23^ and some documents^20,24^. To ensure the effectiveness of the evaluation tool, before the survey, we randomly selected 65 students to do a pre-survey. The Cronbach’s alpha of the survey was 0.881, showing good reliability. The survey consisted of questions designed to assess the following.

#### Tobacco cessation knowledge

There were 30 questions that covered the basic knowledge of effective smoking cessation consultation: 1) tobacco prevalence and harm; 2) cigarettes and nicotine; 3) cigarettes dependence and addiction; 4) adaptive drug therapy for nicotine dependence; 5) the behavior change model at different stages of the smoking cessation process; and 6) counseling strategies about 5As behaviors and 5Rs for smoking cessation. Each correct answer scored 1. The summed score was 1–30.

#### Attitudes towards tobacco cessation

There were 4 questions, with a Cronbach’s alpha of 0.944, involving the perceptions of tobacco cessation importance: 1) nurses should participate in tobacco cessation; 2) nurses thought it was important or not to take additional training or learn skills for tobacco cessation, evaluated on a 1–5 scale from ‘not important at all’ to ‘very important’, and the professional role for tobacco cessation was also evaluated; 3) nurses were non-smoker models; and 4) attitude of nurses towards positive tobacco cessation interventions for patients, evaluated on a 1–5 scale from ‘strongly disagree’ to ‘strongly agree’.

#### Self-efficacy

There were 8 questions with a Cronbach’s alpha of 0.923. Students assessed confidence on 8 aspects about tobacco dependence treatment, e.g. confidence to accurately assess smokers’ willingness to quit smoking, provide tobacco cessation counselling, motivate patients who attempt to quit, and demonstrate the skills for assisting patients throughout the cessation process. The responses ranged from 1 (poor) to 5 (excellent) on a 5-point Likert scale.

#### 5As behaviors

There were 5 questions with a Cronbach’s alpha of 0.933. The 5As behaviors for assisting patients to quit smoking in the clinical practicum were evaluated 3 months post and 6 months post, including the numbers of patients ‘asked about smoking history’, ‘advised to quit’, ‘assessed their readiness to quit’, ‘assisted to quit’ and ‘arranged for follow-up’. The responses were scored 1–5 with 1 = none, 2 = 1–3 patients, 3 = 4–9 patients, 4 = 10–25 patients, and 5 ≥25 patients.

In addition, in order to improve the teaching, teachers collected students’ assessment of the course post the learning process and their experience of tobacco cessation education through an open question.

### Data collection

After having obtained informed consent and before the tobacco cessation education, teachers asked students to complete a baseline survey in the classroom after scanning a QR code. Three months post and 6 months post education, the students were scattered in different hospitals taking a clinical practicum, so it was not possible to perform gathered assessments. Links to Questionnaire Star and informed consent were provided to participants through QQ group or WeChat group of the internship management. Students were offered a small payment to encourage participation to share the 5As behaviors for assisting patients to quit smoking.

### Data analysis

The data were analyzed by SPSS version 20.0 (IBM Corporation, Armonk, NY, USA). The count data were represented by the number of cases (n) and percentage (%). The measurement data were described by mean and standard deviation. Single-arm repeated ANOVA was used to detect the changes of students’ tobacco cessation knowledge, attitude, selfefficacy, and 5As behaviors at different time points pre and post education. A p<0.05 was considered to be statistically significant. The differences that were statistically significant between two time points were analyzed by paired comparison. The Bonferroni method was used for pairwise comparison of the data at two time points for the 285 students who completed the survey at the baseline, 3 months post education, and 6 months post education.

## RESULTS

### Demographics

A total of 572 of the 626 students attending the course completed the baseline survey (91.4%). There were 593 (94.7%) students who provided comments on the course after the learning process; 289 students completed the post education survey at 3 months follow-up ; 348 students completed the post education survey at 6 months; and 285 students completed all three surveys. The students were aged 18–24 years with a mean age of 21.47±0.854 years; 90.5% were female and 2.2% were current smokers.

### Attitudes towards tobacco cessation pre- and post-education assessment

Students reported significant improvement in their knowledge of tobacco cessation after the education and in their perceptions of the importance of ‘nurses should receive or prepare additional tobacco cessation training or skills’ (p<0.05). Understanding of the importance and responsibility of the medical professionals’ involvement in tobacco cessation was not statistically significant (p>0.05). The details are shown in Table 1.

**Table 1:** Knowledge and attitudes pre- and post-education assessments – baseline, 3 months, and 6 months, post education (N=285)

*Items*	*Single-arm repeated ANOVA at differences over time*					*Pairwise comparison p*	
	*Baseline Mean (SD)*	*3 months Mean (SD)*	*6 months Mean (SD)*	*F*	*p*	*3 months/baseline*	*6 months/baseline*
Knowledge	14.66 (3.558)	18.42 (4.420)	19.69 (4.110)	135.542	<0.001	<0.001	<0.001
Importance^‡^							
Tobacco cessation involvement, compared to other disease prevention (nutrition, exercises, etc.)	4.30 (0.982)	4.28 (1.116)	4.34 (1.061)	0.200	0.819	-	-
Nurses obtained additional tobacco control training/skills	4.08 (1.101)	4.29 (1.108)	4.30 (1.003)	3.836	0.022*	0.063	0.038*
Attitudes^†^							
Nurses were non-smoker models	4.20 (1.101)	4.31 (1.154)	4.39 (1.034)	2.099	0.124	-	-
Nurses provided active smoking cessation interventions for patients	4.29 (1.008)	4.34 (1.088)	4.36 (0.956)	0.385	0.681	-	-

P-values were calculated using single-arm repeated (ANOVA) at differences over time.

† 1–5 = ‘strongly disagree’ to ‘strongly agree’.

‡ 1–5=‘ least important’ to ‘most important’.

**p<0.001, *p<0.05.

### Self-efficacy pre- and post-education assessment

Compared with the baseline, the nursing students reported dramatic improvements in their self-efficacy of the 5As behaviors for assisting smokers to quit smoking, 3 months post and 6 months post education. There were no significant differences in the paired comparisons between the baseline and 3 months post education or 6 months post education (p>0.05), except ‘I can provide some simple smoking cessation suggestions for smokers’ (p<0.05). The details are shown in Table 2.

**Table 2:** Self-efficacy pre- and post-education assessments – baseline, 3 months, and 6 months post education (N=285)

*Self-efficacy*	*Single-arm repeated ANOVA at differences over time*					*Pairwise comparison p*	
	*Baseline Mean (SD)*	*3 months Mean (SD)*	*6 months Mean (SD)*	*F*	*p*	*3 months/baseline*	*6 months/baseline*
I can perform accurate assessment of smokers’ tobacco dependence	3.57 (0.903)	3.91 (0.752)	3.90 (0.749)	14.276	<0.001	<0.001	<0.001
I can perform accurate assessment of smokers’ willingness to quit smoking	3.53 (0.829)	3.93 (0.780)	3.95 (0.688)	26.124	<0.001	<0.001	<0.001
I can conclude various risks of smoking according to smokers’ characteristics	3.56 (0.927)	3.04 (0.721)	3.96 (0.706)	20.527	<0.001	<0.001	<0.001
I can conclude various benefits of quit smoking according to smokers’ characteristics	3.79 (0.847)	4.04 (0.718)	4.05 (0.659)	10.096	<0.001	<0.001	<0.001
I can offer some brief quit smoking instructions for smokers	3.97 (0.738)	4.13 (0.680)	4.05 (0.640)	3.526	0.030	0.024	0.438
I can draw up brief quit smoking plans for smokers	3.52 (0.914)	3.92 (0.774)	3.84 (0.743)	17.180	<0.001	<0.001	<0.001
I can arrange appropriate follow-up for smokers	3.57 (0.876)	3.91 (0.802)	3.83 (0.736)	12.593	<0.001	<0.001	<0.001

P-values were calculated using single-arm repeated (ANOVA) at differences over time. Likert scale 1–5 = not confident to very confident. SD: standard deviation.

### Behaviors assisting patients to quit smoking after the tobacco cessation education

The nursing students delivered more 5As behaviors for assisting patients to quit smoking during clinical practicum. The difference in the 5As behaviors between 3 and 6 months post education was statistically significant. The details are shown in Table 3.

**Table 3:** Comparison of the self-reported 5As behaviors for assisting patients to quit smoking, 3 and 6 months post education (N=285)

*Delivery of 5As behavior*	*One-way ANOVA at differences over time*			*Pairwise comparison p*
	*3 months Mean (SD)*	*6 months Mean (SD)*	*F*	*6 months/3 months*
Ask about smoking/tobacco use	2.70 (1.453)	3.19 (1.468)	16.114**	<0.001
Advise patients to quit smoking	2.42 (1.302)	2.96 (1.362)	23.444**	<0.001
Assess readiness to quit smoking	2.21 (1.230)	2.66 (1.392)	16.402**	<0.001
Assist with smoking cessation	2.03 (1.087)	2.51 (1.350)	21.926**	<0.001
Arrange smoking cessation follow-up	1.83 (1.055)	2.27 (1.295)	19.657**	<0.001

The responses were scored 1–5: 1 = none; 2 = 1–3 patients; 3 = 4–9 patients; 4 = 10–25 patients; 5 ≥25 patients. Analyses performed using one-way ANOVA. SD: standard deviation.

** p<0.001.

### Students’ assessments of tobacco cessation education

The students’ views on the tobacco cessation course were collected immediately after the learning process. A total of 593 students shared their opinion, 95.2% (565/593) of the students thought the content was useful and 91.7% (544/593) indicated that the simulation helped them to perform better skills for assisting patients to quit smoking; 92.4% (548/593) of the students perceived the online course materials uploaded by teachers quite suitable for the learning objectives; 89.0% (528/593) of the students stated the course resources were helpful for them to prepare for the simulation; 85.5% (507/593) of the students reported the simulation scenario was realistic; 84.8% (503/593) of the students were very satisfied with the experience of timely support and instruction from the lecturers; 82.5% (489/593) of the students reported well-organized education; 77.1% (457/593) of the students felt well prepared to provide assessment and counselling for patients who were smokers.

In addition, the students shared the following experiences about tobacco cessation learning through an open-ended question, explaining that the training was valuable: ‘I had frequently persuaded my father to quit smoking but I failed as always. This course helped me to know how to make a tailored plan for my father to attempt to quit’; ‘I believe I can apply the skills learned here to solve practical problems’; ‘I learned how to provide specific assistance for patients to quit smoking with the 5As model’; ‘I am confident to provide help to quit smoking to families, relatives and friends’.

The students reported that they implemented smoking cessation skills in practice: ‘I learned a lot of methods and measures of smoking cessation and used them on families’; ‘I learned the theories better and can perform the 5As in practice’; ‘I grasped brief tobacco cessation methods and will apply the skills learned in class to future work’.

The students spoke highly of the various resources in the education. They thought: ‘Online resources are very helpful to improve knowledge and skills about tobacco cessation’; ‘Especially, the video about 5As skills offers direct instructions for scenario simulation, encouraging me to take a good try’; ‘Those scenarios are exactly the same as I encounter in real life’; ‘The scenario simulation helps me to master the 5As interventions of smoking cessation. I can draw up brief plans for smokers to quit and introduce to smokers the harm of smoking and benefits of quitting’; ‘The simulation also helps me to apply specific methods to assist different smokers to quit’. Students said that they ‘would apply that knowledge and perform the skills learned here in clinical practicum’.

## DISCUSSION

This study suggests that the inclusion of tobacco cessation education, directed by the Guidelines^5^ and the Manual^23^, into nursing compulsory courses, in the form of a classroom didactic session, online selflearning, and a scenario simulation session, effectively increased nursing students’ knowledge, skills, and confidence in tobacco cessation, which was consistent with the results of previous studies1^6–19^. This study also found that students performed skills learned in the course to actively assist patients to quit smoking. These findings indicate that it is feasible and practical to promote tobacco cessation knowledge and skills for all nursing students and to increase the students’ behaviors for assisting patients to quit smoking.

The tobacco cessation education achieved relatively good results, probably because of the traditional didactic session with specific instruction on tobacco cessation health education theories such as the transtheoretical model of behavior change^25^, the 5As and 5Rs theoretical models of tobacco cessation, and the provision of the corresponding online resources. Those online resources were about different cases with assistance from different medical professionals, which were easily accessible and could be repeatedly learned^17–19^. The online Q and A helped students to solve problems in self-learning. The teachers supervised and checked the process and results of the self-learning online. Students’ self-learning was scored using the formative assessment for the quantity, duration, and test result. Additionally, the scenario simulations of 5As behaviors provided the opportunity for experimental learning^16,17,19,26,27^. The scenario cases were similar to those encountered by nurses during daily work, facilitating nursing students to apply knowledge into practice and to develop their problem-solving competency. The simulation offered students realistic scenarios to role play and experience real situations, enhancing 5As behavior skills by exploring communication and interaction with various clients and doing specific practice. This blended online and offline teaching gave students novel experiences and was favored by students^17,19,27^.

The tobacco cessation education did not affect the students’ role identification, likely because of the positive attitudes before the education. Chinese nursing students were mostly female and few were smokers; thus, they had very positive attitudes towards smoking cessation^28^.

Previous studies have indicated that teaching resources for tobacco cessation education have been held back^29^. This study designed content and developed resources from the Guidelines^5^ and the Manual^23^, which also provided free online resources on their websites. Community Nursing is a compulsory course for baccalaureate students. It explicitly requires students to master health education skills and to promote health. All Chinese schools can access these resources and integrate tobacco education into compulsory courses, and therefore enhance the corresponding knowledge and skills and help more patients to quit smoking. It can also help to achieve the goal of decreasing the current smoking rate of 27.7% to 20% or lower the smoking population aged >15 years, by 2030^30^.

To the best of our knowledge, this is the first study to integrate tobacco cessation education into a compulsory course in China. The results indicate important information for nursing schools, i.e. smoking cessation skills can be effectively improved in a clinic setting with no additional class time. Tobacco cessation education should be promoted for nursing students to develop more professionals for assisting with tobacco cessation.

### Limitations

This study has some limitations. First, it was a onegroup pre- and post-intervention design rather than a controlled trial design; therefore, it could not manage the interference of confounders. In the future, we should conduct controlled trial research. Second, the online response rate was quite low. Because the students were scattered in different hospitals taking their practicum, the data were collected through a QQ group or Wechat group of internship management, but not centrally, 3 and 6 months post education, with 50.5% and 60.8%, respectively, fewer collected than those centrally collected at the time of the baseline survey. There were only 285 (49.8%) students who completed all three surveys, and only these surveys were analyzed. Surveys from students completing less than three surveys were not included in the data analysis, which may have caused a deviation. Third, the study only compared the vertical change of the 5As behaviors for assisting patients to quit smoking, because students still studied on campus and did not serve patients at the time of the baseline survey. Fourth, the online self-reported changes instead of direct observation of 5As behaviors or reading medical records might be incorrect, more or less, which is an inherent limitation of an online survey. Future studies should be conducted on direct tracking of tobacco cessation interventions. Fifth, students’ knowledge was less than 70% post education. One reason was that it was tested three months later, rather than immediately. Finally, this study was implemented only in one university in Chongqing, China, and the effectiveness might not be nationally representative. Further studies in more universities and among different education degrees are needed.

## CONCLUSIONS

The integration of tobacco cessation education into compulsory courses could enhance and improve nursing students’ clinical knowledge and skills, and also improve clinical skills for assisting patients to quit smoking.

## Data Availability

The data supporting this research is available from the authors on reasonable request.
